# Appetite for destruction: the inhibition of glycolysis as a therapy for tuberous sclerosis complex-related tumors

**DOI:** 10.1186/1741-7007-9-69

**Published:** 2011-10-21

**Authors:** Alfredo Csibi, John Blenis

**Affiliations:** 1 Department of Cell Biology, Harvard Medical School, 240 Longwood Ave, Boston, MA 02115, USA

## Abstract

The elevated metabolic requirements of cancer cells reflect their rapid growth and proliferation and are met through mutations in oncogenes and tumor suppressor genes that reprogram cellular processes. For example, in tuberous sclerosis complex (TSC)-related tumors, the loss of *TSC1/2 *function causes constitutive mTORC1 activity, which stimulates glycolysis, resulting in glucose addiction *in vitro*. In research published in *Cell and Bioscience*, Jiang and colleagues show that pharmacological restriction of glucose metabolism decreases tumor progression in a TSC xenograft model.

See research article: http://www.cellandbioscience.com/content/1/1/34

## Commentary

The utilization of nutrients from the environment by normal cells is controlled by fine-tuned mechanisms regulated by growth factor signals. Cancer cells overcome this growth factor dependence by acquiring genetic mutations that rewire signaling pathways that affect the uptake of nutrients, and reprogram metabolism to fuel the biosynthetic processes required to support their altered cell growth, survival and proliferation [1]. This rewiring for growth may make tumors more vulnerable to nutrient deprivation, however. For instance, growing evidence demonstrates that cancer-promoting mutations result in addiction to nutrients, particularly glucose. In an article in *Cell and Bioscience*, Jiang *et al. *[2] explore the therapeutic potential of glucose deprivation, by both pharmacological and dietary means, in a rodent model of tuberous sclerosis complex-related tumors, which display glucose addiction *in vitro*.

## The glucose appetite of tumors and its regulation by mTORC1

Research efforts have sought to characterize tumor cell metabolism since Otto Warburg's observations in the 1920s of the tendency of cancer cells to metabolize glucose into lactate despite sufficient oxygen levels (known as the Warburg effect or 'aerobic glycolysis'). By contrast, most differentiated cells primarily metabolize glucose to carbon dioxide by oxidation of pyruvate in the mitochondrial tricarboxylic acid (TCA) cycle, a process known as oxidative phosphorylation that requires far less glucose to generate the same amount of energy. The heightened appetite of tumor cells for glucose has been put to diagnostic use, as high rates of glucose utilization can be detected using [^18^F]-fluorodeoxyglucose positron emission tomography (FDG-PET), providing images in which tumors often appear as PET-positive as the most metabolically active tissues. Understanding why and how the Warburg effect occurs has posed a puzzle, however, as it is not immediately obvious why aerobic glycolysis should be favored in tumors when it is an inefficient way to generate energy. It is thought that the Warburg effect supports tumor growth by diverting glucose to macromolecular precursors, such as acetyl-coA for fatty acids, glycolytic intermediates for nonessential amino acids, and ribose for nucleotides [3].

**Figure 1:**
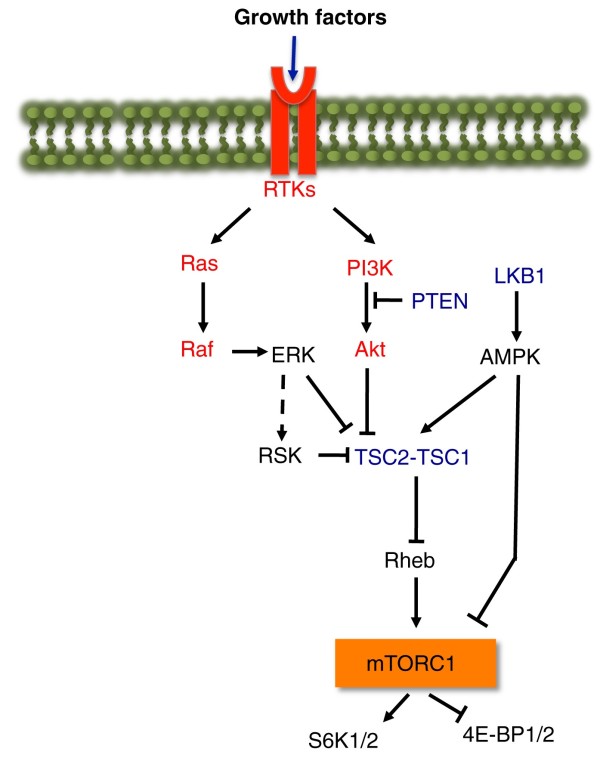
**A network of oncogenes and tumor suppressors regulates the mTORC1-signaling pathway**. Growth factors bind and stimulate receptor tyrosine kinases (RTKs), which can activate both the PI3K-Akt and Ras-ERK signaling pathways. These upstream signals inhibit the TSC1-TSC2 complex allowing Rheb to activate mTORC1. Activated mTORC1 phosphorylates two direct substrates, the ribosomal S6 kinases (S6K1 and S6K2), and translation repressors 4E-BP1 and 4E-BP2. Cellular energy depletion results in the activation of AMP-activated protein kinase (AMPK) by the tumor suppressor protein LKB1 serine/threonine kinase. AMPK phosphorylates and enhances the GAP function of TSC2 towards Rheb. In addition, AMPK directly phosphorylates the mTORC1 component raptor. Both events result in the inhibition of mTORC1 in response to energy stress. Within this signaling network lie many oncogenes (depicted in red) and tumor suppressors (depicted in blue).

**Figure 2:**
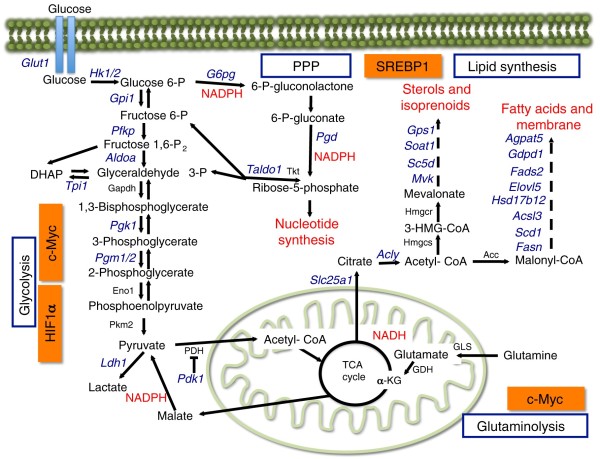
**The mTORC1 pathway controls cellular metabolism**. The mTORC1 signaling pathway controls metabolic pathways active in proliferating cells. This schematic shows our current understanding of how glycolysis, oxidative phosphorylation, the pentose phosphate pathway (PPP), and glutamine metabolism are interconnected in proliferating cells. The mTORC1 pathway participates in this metabolic rewiring by controlling the expression of genes (depicted in blue) encoding enzymes involved in glycolysis, the PPP, and lipid synthesis. This metabolic control requires the up-regulation of c-Myc and HIF1α (glycolysis), and SREBP1 (lipid biosynthesis and the PPP). mTORC1-dependent metabolic regulation allows for production of both NADPH and intermediates for macromolecular synthesis (depicted in red). c-Myc drives glutamine metabolism, which also supports NADH production.

In normal cells the switch from a non-proliferating state, in which oxidative phosphorylation meets the cell's energy needs, to proliferation, in which glycolysis dominates, is triggered by growth factors acting through the mammalian Target of Rapamycin Complex 1 (mTORC1) signaling pathway (Figure 1). This pathway allows cells to integrate information about environmental conditions and to balance catabolic and anabolic processes accordingly. Growth factor-activated kinases phosphorylate and inhibit the tumor sclerosis complex TSC1-TSC2, allowing the small G protein Rheb to activate mTORC1. In addition, mTORC1 is sensitive to intracellular energy levels through the AMP-activated protein kinase (AMPK). In response to energy deprivation, AMPK phosphorylates TSC2, and the mTORC1 component raptor, resulting in mTORC1 inhibition and a reduction of energy consumption [4] (Figure 1). The most recognized function for mTORC1 is the promotion of protein synthesis through the phosphorylation of at least two direct downstream targets, the ribosomal S6 kinases (S6K1 and S6K2), and the translation repressors eIF4E-binding proteins 1 and 2 (4E-BP1 and 4E-BP2) [4] (Figure 1). However, studies using the mTORC1-specific inhibitor rapamycin have revealed a broader role of mTORC1 in regulating the metabolic processes that support cell growth and proliferation (Figure 2).

In addition to driving protein synthesis, activation of mTORC1 promotes glycolysis by up-regulating Hypoxia-inducible factor (HIF1α) and c-Myc, which in turn promote the expression of proteins involved in nearly every step of the glycolytic pathway. mTORC1 activation also contributes to the Warburg effect by diverting pyruvate away from oxidation in mitochondria. This is achieved by increased expression of lactate dehydrogenase, which converts pyruvate to lactate, and pyruvate dehydrogenase kinase, which inhibits the conversion of pyruvate to acetyl-CoA. Furthermore, mTORC1 stimulates *de novo *lipogenesis by promoting the expression of genes regulated by the sterol regulatory element-binding protein (SREBP1), thereby promoting the synthesis of the fatty acids needed for new cell membranes. Interestingly, mTORC1 activation is also sufficient to stimulate the expression of genes encoding the enzymes involved in the pentose phosphate pathway (PPP), including glucose 6-phosphate dehydrogenase (G6PD) in an SREBP1-dependent manner. Activation of the PPP provides cells with sources of NADPH to drive anabolic metabolism, as well as the generation of ribose 5-phosphate for *de novo *nucleotide biosynthesis [5] (Figure 2). It remains to be determined whether the mTORC1 pathway influences the metabolism of glutamine (glutaminolysis), an abundant nutrient essential for cancer cell bioenergetics and proliferation. It is known that c-Myc can stimulate glutamine catabolism [6], however, suggesting that mTORC1-dependent regulation of c-Myc promotes glutaminolysis as well as glycolysis.

In sum, the various metabolic outcomes of activating the mTORC1 pathway render cells dependent on glucose and perhaps glutamine to support their biosynthetic needs for rapid growth and proliferation. In normal cells, mTORC1 activation is regulated in accordance with the needs of the organism, but in cancer cells, mutations in various oncogenes or tumor suppressors can lead to its aberrant activation (Figure 1). In TSC-related tumors the loss of the TSC1/2 complex leads to mTORC1 activation irrespective of growth factor or energy levels, and also to reduced insulin-stimulated glucose uptake [7], resulting in an impaired ability to balance metabolic demand with supply. Whether the consequent hypersensitivity of TSC-related tumors to glucose deprivation that is observed *in vitro *[8] can be exploited therapeutically *in vivo *is addressed by the study of Jiang *et al. *[2].

## The glucose addiction of *TSC*-mutant tumors as a therapeutic target

TSC is an autosomal dominant genetic disease with an incidence of 1 in 6,000 at birth, and nearly 1,000,000 people worldwide are known to have TSC. TSC is due to genetic inheritance or spontaneous inactivating mutations in either *TSC1 *or *TSC2 *genes, and is characterized by the formation of non-invasive benign tumors in many organs.

Rapamycin and its analogues, because of their inhibitory effect on the mTORC1 pathway, have been examined as potential therapeutic agents in the treatment of TSC; however, early studies have demonstrated that while these drugs can reduce tumor size, the tumors return after treatment stops [9]. Therefore, identifying new therapeutic options that can specifically eliminate TSC tumors remains an important goal. Targeting cellular metabolism has received particular attention during the past few years as an alternative strategy for cancer therapy, and could prove an important approach for treating TSC based on the rationale that *TSC*1/2-/- cells require glucose for survival [8].

The research of Jiang *et al. *[2] sought to examine the effects of the glycolytic inhibitor 2-deoxy-D-glucose (2-DG) and a diet free of carbohydrates on the growth of LEF2 cells from a *Tsc2*-null rat tumor implanted in mice. The exposure of these cells to 2-DG resulted in decreased cell viability at low glucose concentration. 2-DG is an analog of glucose in which the 2-hydroxyl group has been replaced by hydrogen, thus preventing it from undergoing glycolysis. This leads to reduced cellular ATP levels and subsequently cell growth. Jiang *et al. *show that 2-DG treatment suppresses tumor growth by reducing cell proliferation in this model, although they did not show whether this is accompanied by apoptosis as occurs *in vitro *[2]. These observations suggest that 2-DG is a promising antitumor therapy and, in fact, this compound is being used to treat osteosarcomas and lung cancers in phase II clinical trials. However, pre-trial studies show that 2-DG, as is the case with other glycolysis inhibitors, does not have a significant effect on tumor growth when used on its own as a monotherapy, although it can sensitize tumors to chemotherapeutic agents such as paclitaxel [10]. The differences between previous studies and the results shown by Jiang *et al. *raise the question of whether 2-DG effects are dependent on the type of tumor, and if this could be specifically related to the hypersensitivity of *TSC2*-/- tumors to glucose deprivation.

Jiang *et al. *also tested the effects of a diet free of carbohydrates in their model, anticipating that this would also deprive the *TSC2*-/- tumors of glucose, and add to the effects of 2-DG. However their diet (which was carbohydrate-free but not calorie-restricted) produced some unexpected results. In humans a carbohydrate-free diet leads to reduced blood glucose and an increased production of ketone bodies as nutrients other than glucose are used to produce energy [11], but in the mice fed with a carb-free diet in the study of Jiang *et al. *levels of β-hydroxybutyrate (HOB), a ketoacid, were not affected and blood glucose remained high [2]. Strikingly, and in contrast to the results of 2-DG treatment, this diet resulted in larger tumors with increased necrosis and zones of liquefaction. These large tumors did not appear to be fueled by glucose, however, as the diet was effective in decreasing the uptake of [^18^F]-fluorodeoxyglucose (FDG), suggesting that nutrient sources other than glucose are fuelling anabolism and survival under these conditions [2].

The carb-free diet provides abundant fatty acids that are broken down by beta-oxidation into acetyl-CoA, a major substrate for energy and biomass production through the TCA cycle. Interestingly, a recent report demonstrates that high-grade primary tumors contain elevated levels of fatty acids that contribute to the proliferation of aggressive cancer cells by increasing the levels of signaling lipids such as phosphatidic acid, lysophosphatidic acid and prostaglandin E_2 _[12]. Consistent with this, Jiang *et al. *[2] show that treatment of LEF2 cells with oleic acid results in increased proliferation and survival. In contrast, treatment with the saturated palmitic acid induces apoptosis in LEF2 cells, an observation that might be relevant to the increased areas of necrosis observed in the tumors of carb-free fed mice [2].

While the carb-free diet failed to inhibit TSC tumor progression, the 2-DG effects on tumor growth are promising and encouraging for future clinical trials in TSC patients. However, toxicity due to off-target effects has been attributed to this compound in clinical trials, and three of ten mice in this study were sacrificed early on account of weight loss during 2-DG treatment [2], suggesting that there are inherent difficulties in an approach that attempts to starve a tumor but not the organism that hosts it. One way of overcoming toxicity problems while improving efficacy is to use combination therapies, and for TSC-related tumors there are good reasons to consider these in future studies. TSC tumors display low FDG uptake on PET imaging despite increased glycolytic flux [13], suggesting that a glucose-independent nutrient source is fueling the cells. Moreover, recent work has demonstrated that glutamine is required to maintain the cellular bioenergetics of *TSC*-/- cells [14]. Therefore combination therapies targeting both glutamine and glucose addiction might be effective.

Cancer therapy is increasingly shifting toward individualized therapeutic approaches based on the genetic abnormalities exhibited by transformed cells. Jiang *et al. *[2] demonstrate that targeting glucose addiction is an effective approach for decreasing the growth of tumors driven by TSC mutations. Thus glucose addiction may prove to be the 'Achilles' heel' for the treatment of TSC. Whether these findings will translate to other tumor types, in which the constitutive activation of mTORC1 is a result of different genetic abnormalities, and whether the toxic side effects of 2-DG can be overcome, however, remains to be seen.
